# On the Hysterotome for the Cure of Dysmenorrhea, Invented by Dr. O. A. White, of Charleston, S. C.

**Published:** 1859-08

**Authors:** 


### ON THE HYSTEROTOME FOR THE CURE OF DYSMENORRHEA, INVENTED BY DR. O. A. WHITE, OF CHARLESTON, S. C.

[From the *Charleston Medical Journal and Review.*]

The author of this article assumes the cause of Dysmenorrhea to be a "preternatural narrowing of the uterine canal," which in some cases is doubtless true, and it then generally depends upon defective development of the neck of the uterus. He then proceeds to recommend in these cases, as a means of cure, incisions within the neck of the uterus, which, he claims, can be most easily and efficiently made by the use of his hysterotome, which is an instrument similar to a male catheter, that can be readily introduced to the desired extent, and then by means of a screw, two blades can be made to project from opposite sides, which make the incisions as the instrument is withdrawn. After which, by the use of sponge tents for a short time, the part is kept dilated.

Now, is it philosophical to treat a part in such a condition by incisions? the first effect of which would be to abstract a certain amount of blood from the part in which there is already a defective circulation. Would we treat any other atrophied part in this way? On the contrary, the plan suggested long ago by Dr. McIntosh is doubtless the correct one, which is, to carefully and repeatedly dilate the os and cervix uteri with bougies of various sizes. This is in accordance with the principle well understood by every tyro in the profession, viz., that a part is increased in size and power by exercise. Besides the plan of treatment, proposed in the paper, for these cases, must be injurious on another account, the cicatrices which would necessarily follow the incisions, would, by contracting, as they always do, render still narrower the passage already too nearly closed, and the author's attempt to meet this objection by the temporary use of sponge tents, must fail, for the contraction of a cicatrix continues even after the tents are withdrawn. And again, should conception follow these operations, as it sometimes does, another difficulty might be reasonably apprehended, as a consequence of the cicatrices, viz., an unyielding neck and os uteri.

Dysmenorrhea may depend upon endo-metritis, and when it does, this instrument may be made useful, in abstracting blood from the part affected, upon the principle of local depletion, but such cases are rare, and form but a small proportion of the cases where narrowing of the neck of the uterus exists.

It was not our intention to specify all the causes of dysmenorrhea at this time, but we will allude to one which has become quite prevalent of late. Ladies who obtain their knowledge of hygiene from hydropathic journals, and are in the habit of daily cold bathing, are commonly the subjects of dysmenorrhea, and for these we have frequently been called to prescribe.

Believing that in these cases the painful menstruation was caused by congestion of the uterus, or its lining membrane, which was kept up by the repeated application of cold to the surface, the enfeebled organ not being able to react as a healthy structure would do, we have not unfrequently been gratified to find that all the painful sensations have disappeared upon our prescribing the cold bath.

In cases like those referred to in the paper which has called out these remarks, there are good reasons, as well as high authority, for the teaching that all rudeness and violence should be avoided.

